# Supplementary material for: In Vivo Trafficking of the Anticancer Drug Tris(8-Quinolinolato) Gallium (III) (KP46) by Gallium-68/67 PET/SPECT Imaging
Source: Molecules. 2023 Oct 22;28(20):7217. doi: 10.3390/molecules28207217 (PMC10609081; doi:10.3390/molecules28207217)
Supplement: Supplementary file 1 [file molecules-28-07217-s001.zip › molecules-2670253-supplementary.pdf]

In vivo trafficking of the anti-cancer drug tris(8-quinolinolato) gallium (III) (KP46) by gallium-68/67 PET/SPECT imaging

Afnan M F Darwesh, Cinzia Imberti, Joanna J Bartnicka, Fahad Al-Salamee, Julia E Blower, Alex Rigby, Jayanta Bordoloi, Alex Griffiths, Michelle Ma and Philip J Blower

Supplementary Materials

Chemical and radiochemical characterisation

LC/MS

KP46 and 8-hydroxyquinoline (control) were dissolved in ethanol and diluted with water to bring the final concentration to 250/350 µM. Mobile phase: H_2O (A) and acetonitrile (B). Gradient: 0–2 minutes: 5% B, 2–17 minutes: 60% B, 17–18 minutes: 100% B, 18–24 minutes: 100% B, 24–26 minutes: 5% B and 26–30 minutes: 5% B. LC/MS analysis (Fig. S1) of 8-hydroxyquinoline showed a single HPLC peak at 14.05 min, which corresponded to the ligand mass seen in the mass spectrometry analysis at 146 m/z $[M+H]^+$. LC/MS analysis (Fig. S1) of KP46 showed one peak eluting at 16.0 min, which gave a molecular ion in the mass spectrometry analysis at 502 $[M+H]^+$ (showing the isotopic pattern consistent with a mononuclear gallium complex $[Ga(8HQ)_3]H^+$). Other ions shown in Fig. S1 corresponding to gallium-8HQ complexes were also seen in Fig. S3 (m/z = 357 ($Ga(8HQ)_2^+$), 1005 ($Ga_2(8HQ)_6H^+$) and another at 398 (not seen in Fig. S3, consistent with $[Ga(8HQ)_2(CH_3CN)]^+$. Another HPLC peak eluting at 14.0 min in the chromatogram corresponded to the free ligand 8-hydroxyquinoline in both its elution time and mass spectrum (Fig. S1). Since free 8-hydroxyquinoline was not detected in the ^1H NMR spectrum of KP46, the free ligand detected by LC-MS must have been formed by dissociation during the HPLC elution, resulting in both free ligand (eluting ahead of the gallium complexes in the range 14-16 min) and gallium complexes with reduced 8HQ:Ga ratio eluting later. These observations are consistent with dissociative equilibria discussed in the main text, with the extent of dissociation increased during HPLC as the spatial separation of free 8-hydroxyquinoline from gallium complexes drives the dissociative reaction further than in the parent solution (where dissociation is undetectable by ^1H NMR).

NMR analysis

^1H NMR of 8-hydroxyquinoline: (DMSO-d^6 400 MHz) δ: 8.84 (dd, *J* = 4.1, 1.5 H_Z, 1H), 8.30 (dd, *J* = 8.3, 1.5 H_Z, 1H), 7.52 (dd, *J* = 8.3, 4.1 H_Z, 1H), 7.43 (t, *J* = 8.0 H_Z, 1H), 7.38 (dd, *J* = 8.1, 1.1 H_Z, 1H), 7.11 (dd, *J* = 7.4, 1.2 H_Z, 1H).

^1H NMR of KP46: (DMSO-d^6 400 MHz) δ: 8.71 (0.63 H), 8.57 (1H), 7.65 (0.68 H), 7.50 (1.67 H), 7.18 (1H), 6.88 (1H). In addition to peaks assignable to 8HQ ligand protons, additional peaks were found at 1.9 ppm (assigned to acetic acid methyl protons), 2.5 ppm (DMSO-d^5) and 3.4 ppm (H_2O).

Figure S2 (below) shows the NMR spectra of 8-hydroxyquinoline and the sample of KP46. The assignments shown for KP46 are based on the COSY spectrum also shown in Figure S2. The KP46 spectrum shows the absence of free ligand in the sample. However, signals of non-equivalent ligand protons were detected indicating the possible coexistent of KP46 structural isomers in the solution (e.g. *fac*- and *mer*-isomers).

Elemental analysis

Found: C 61.5%, H 3.3%, N 7.6%; calc. for tris(hydroxyquinolinato)gallium(III) with one molecule of acetic acid of crystallisation, as observed by ^1H NMR ($C_{29}H_{22}N_3O_5Ga$): C 61.9%, H 3.9%, N 7.5%.

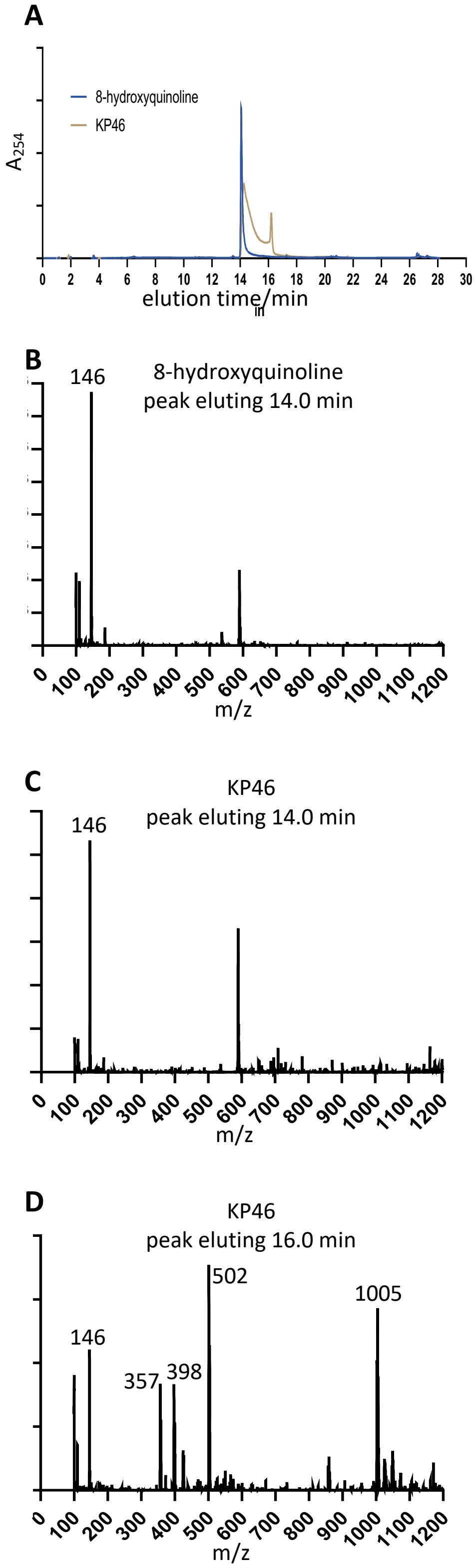

Figure S1. LC/MS analysis of 8-hydroxyquinoline and KP46. **A**: Reverse phase HPLC chromatograms of 8-hydroxyquinoline (blue, eluting 14.0 min) and KP46 (brown, with peaks eluting from 14.0 to 16.0 min). **B**: Electrospray mass spectrometry of the fraction eluting at 14.0 min during HPLC of 8-hydroxyquinoline, showing molecular ion at m/z = 146. **C**: Electrospray mass spectrometry of the fraction eluting at 14.0 min during HPLC of KP46, showing similar spectrum to that of 8-hydroxyquinoline and hence indicating that KP46 partially dissociates during chromatographic separation. **D**: Electrospray mass spectrometry of the fraction eluting at 16 min during HPLC of KP46, showing the presence of $[Ga(8HQ)_3]H^+$ (m/z = 502) and other Ga-8HQ species (see Fig. S3 for assignments).

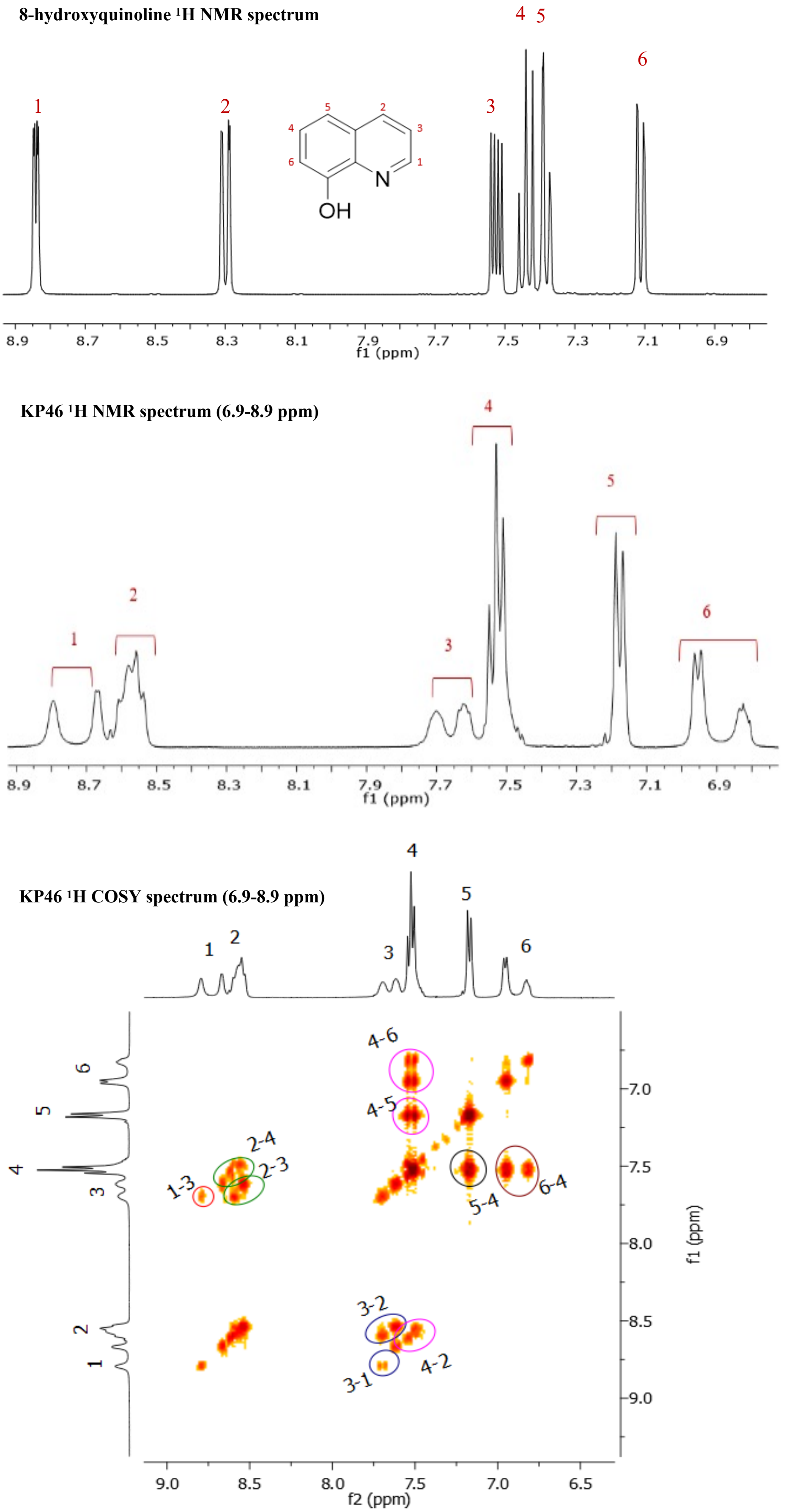

Figure S2. ^{1}H NMR and COSY spectra of 8-hydroxyquinoline and KP46

High resolution mass spectrometry

Samples of KP46 were dissolved in ethanol and diluted 10-fold with 0.1% formic acid in 50% methanol for injection into the electrospray mass spectrometer. Mass spectrometry (Fig. S3) showed a peak at *m/z* 502.0677 (calculated: 502.0682 for $C_{27}H_{18}GaN_3O_3 + H^+$) corresponding to KP46 ([Ga(8HQ)$_3$]) in the cation form of $M+H^+$ for the ^{69}Ga isotope. An expansion of this peak envelope is shown in Fig. S3. This molecular ion, corresponding to $[Ga(8HQ)_3]+H^+$, was however not the most abundant ion in the electrospray mass spectrum, most likely because it acquires its positive charge by protonation of KP46, which is inefficient as KP46 has no basic sites. Other species were detected in greater abundance including $[Ga(8HQ)_2]^+$ (found: 357.0148; calculated: 357.0155 for $C_{18}H_{12}GaN_2O_2$) which acquires its positive charge by loss of one hydroxyquinolate ligand from KP46; $[Ga_2(8HQ)_4(OH)]^+$ (found: 733.0324; calculated for $C_{36}H_{25}Ga_2N_4O_5$: 733.03276); $[Ga_2(8HQ)_4CHO_2]^+$ where the CHO_2 fragment originates from formate (found: 761.0273; calculated for $C_{37}H_{25}Ga_2N_4O_6$: 761.0277) formed in the formate-containing injection solution or during the ionisation process; $[Ga_2(8HQ)_5]^+$ (found: 860.0750; calculated for $C_{45}H_{30}Ga_2N_5O_5$: 860.07450); $[Ga_2(8HQ)_6]H^+$ (found: 1005.1287; calculated for $C_{54}H_{37}Ga_2N_6O_6$: 1005.1329). Since the low 8HQ:Ga ratio in several of the observed ions is not consistent with elemental analysis data on the solid samples of KP46, these additional peaks may arise via solution equilibria in the injection solution or by dissociation, dimerisation and hydrolysis reactions occurring during the electrospray ionisation process.

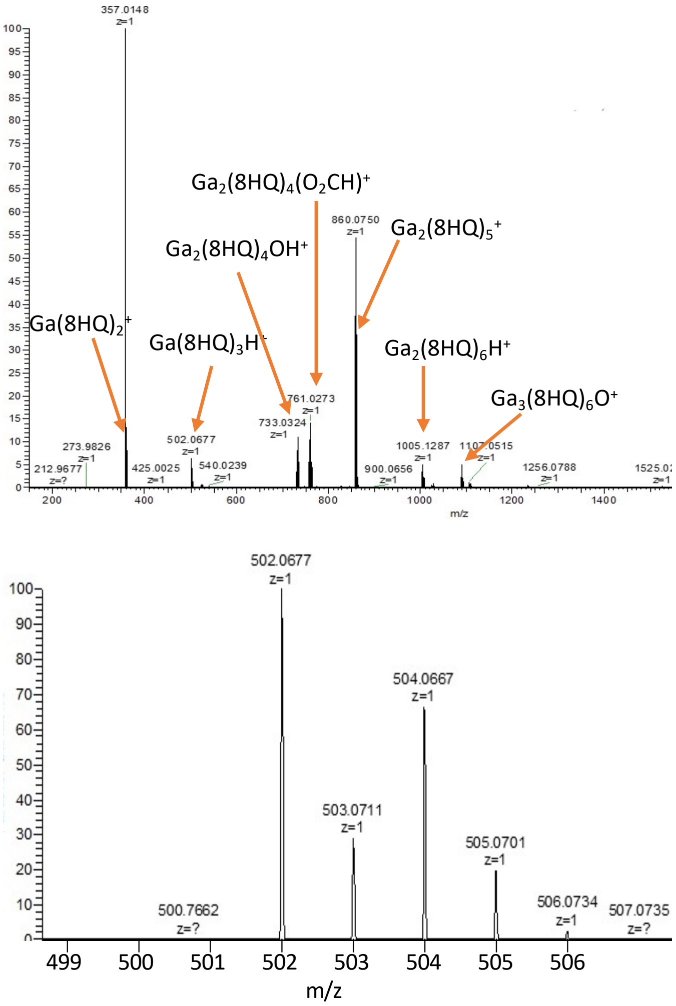

Figure S3. Electrospray mass spectrum of KP46 sample (top) and expansion of the envelope corresponding to $[Ga(8HQ)_3]+H^+$ (bottom).

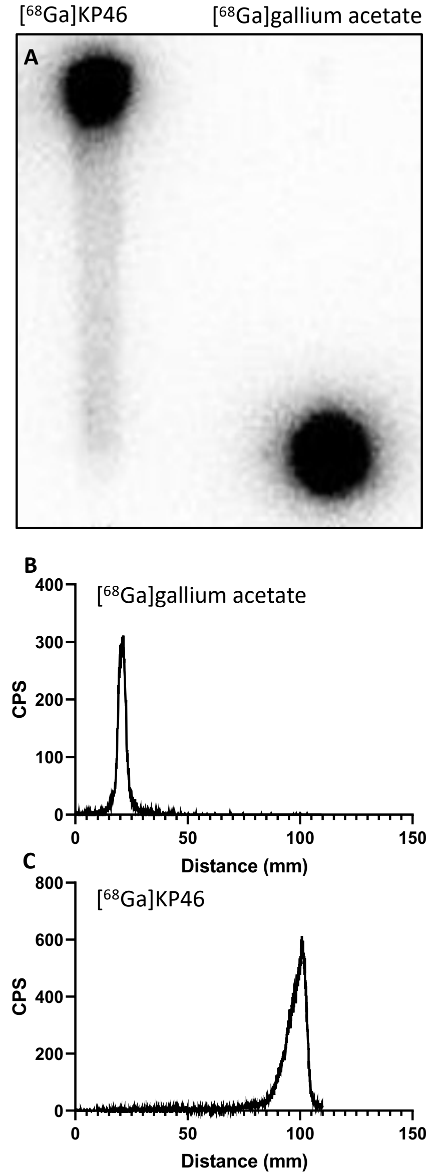

Figure S4. Instant thin layer chromatography (iTLC) on silica gel impregnated glass microfiber strips of [^{68}Ga]gallium acetate and [^{68}Ga]KP46. **A**: Radioactivity distribution imaged using phosphor imager; **B**: [^{68}Ga]gallium acetate iTLC (Rf = 0) analysed by Lablogic mini scan TLC reader with β^{+} detection; **C**: [^{68}Ga]KP46 iTLC (Rf = 1) analysed by Lablogic mini scan TLC reader with β^{+} detection.

Chromatography and radiochromatography

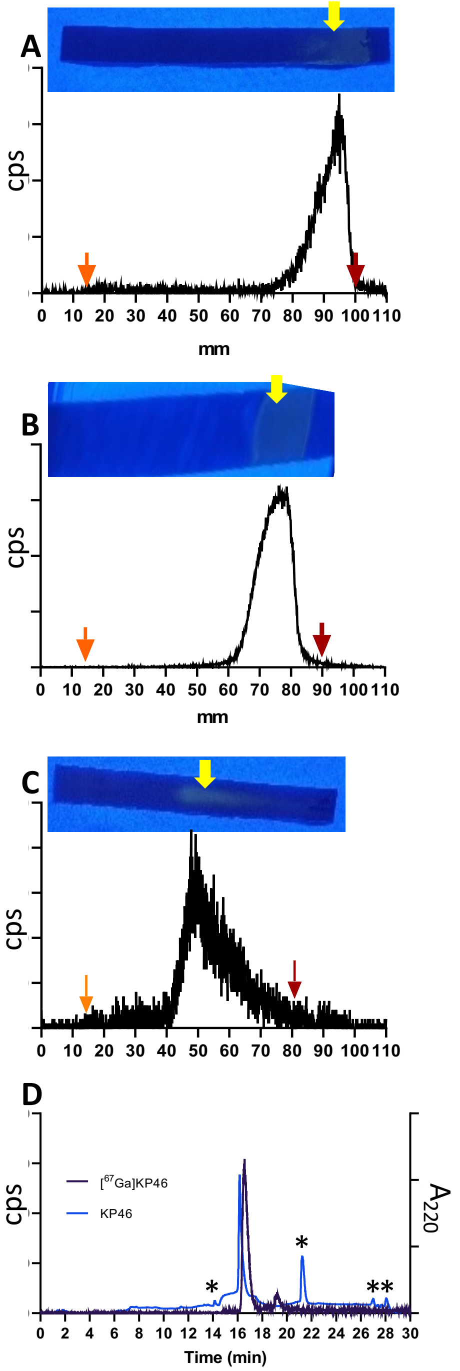

Figure S5. iTLC analysis of KP46 and [$^{68/67}$Ga]KP46 showing indistinguishable chromatographic properties of radioactive and non-radioactive forms. **A**, **B** and **C** each show a photograph under UV illumination (top) and radioactivity distribution (bottom) of iTLC strip after chromatographic development of a spot of KP46 (dissolved in DMSO) mixed with ethanol solution of [^{67}Ga]KP46 (**A**) and [^{68}Ga]KP46 (**B**), all with Rf = 0.8-0.1. **C** shows the KP46/[^{67}Ga]KP46 sample mixed with vehicle (water/PEG), in which the Rf value of both KP46 and [^{67}Ga]KP46 is reduced to approx. 0.55. Red arrows in radioactive scans show position of solvent front, orange arrows show position of origin, and yellow arrows indicate the UV-active spot in each photograph. **D** shows HPLC chromatogram, with UV (220 nm, blue line) and radioactive (black line) detection, of KP47 mixed with [^{67}Ga]KP46. The 30-second delay between the principal corresponding UV and radioactive peaks corresponds to the transit time from UV to radioactivity detector. UV-peaks labelled with asterisks are artifacts present in blank samples and not components of the sample.

Lipophilicity measurement

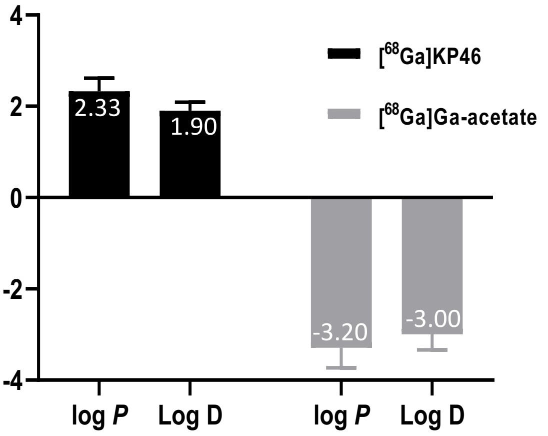

Figure S6. Log$D_{(octanol/PBS)}$ and log$P_{(octanol/water)}$ analysis of [^{68}Ga]KP46 and [^{68}Ga]Ga-acetate (control) using shake flask method. Results are reported as mean ± SD.

Serum protein binding - controls

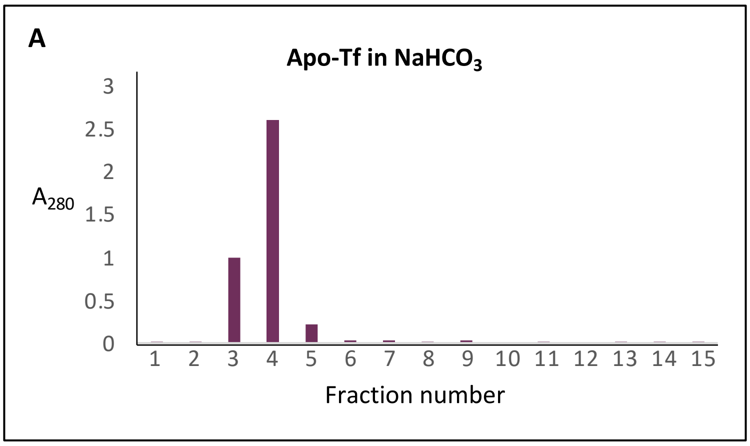

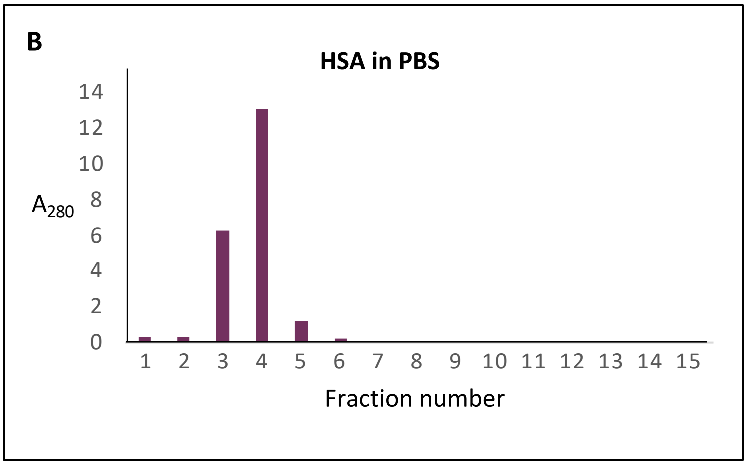

Figure S7. Size exclusion elution profile of apo-Tf in $NaHCO_3$ (**A**) and HSA in PBS (**B**). Absorbance of each fraction at 280 nm was measured using nanodrop spectrophotometer. Each fraction is 0.5 ml.

group	A	A	B	B	C	C	D	D	E	E	F	F	2 h	2 h
	%ID/g	SD %ID/g	%ID/g	SD %ID/g	%ID/g	SD %ID/g	%ID/g	SD %ID/g	%ID/g	SD %ID/g	%ID/g	SD %ID/g	%ID/g	SD %ID/g
tumour	6.10	2.40	2.62	0.86	0.16	1.10	0.47	0.29	0.31	0.12	1.57	0.48	3.24	0.39
skin/fur	3.73	0.69	1.45	0.48	0.08	0.16	0.07	0.07	0.03	0.06	0.23	0.10	2.55	0.23
muscle	1.04	0.41	1.24	0.28	0.08	0.16	0.01	0.01	0.03	0.02	0.11	0.10	3.01	0.23
femur	15.54	6.70	5.39	1.04	0.47	1.26	0.29	0.73	0.19	0.12	2.55	1.82	7.03	0.54
bladder	5.52	4.14	3.52	2.00	0.08	0.47	0.07	0.58	0.02	0.09	0.35	0.18	3.78	0.69
blood	7.39	3.38	5.39	0.62	1.73	1.57	0.07	1.46	0.02	0.08	0.13	0.04	9.96	0.31
SI	6.84	2.07	13.88	10.70	12.75	4.14	18.36	4.95	0.33	0.08	0.82	0.61	11.97	1.62
LI	2.56	0.76	4.07	1.10	61.66	21.54	56.54	21.44	0.59	0.62	0.62	0.47	2.78	0.46
stomach	1.38	0.69	2.83	0.55	11.02	10.08	11.90	8.14	0.70	1.07	0.42	0.14	3.09	1.70
pancreas	1.93	2.97	7.32	2.62	0.16	0.79	0.06	0.15	0.11	0.08	0.35	0.23	7.57	0.46
spleen	4.14	3.11	6.22	2.56	0.16	0.94	0.06	0.15	0.03	0.06	0.55	0.38	7.72	0.69
Lkidney	8.29	3.04	14.36	2.97	0.79	0.94	0.44	0.87	0.11	0.09	0.44	0.28	15.75	0.77
Rkidney	8.22	2.07	13.81	3.31	1.10	1.26	0.44	0.87	0.12	0.11	0.55	0.27	14.59	2.01
liver	8.29	1.59	26.86	4.14	4.72	3.15	1.75	1.75	0.39	0.48	1.34	1.01	31.58	3.78
heart	1.93	0.76	15.19	2.76	0.31	1.26	0.07	0.29	0.03	0.05	0.35	0.27	12.59	1.62
lungs	5.46	1.73	11.67	3.80	0.47	1.10	0.15	0.15	0.16	0.40	0.41	0.34	14.29	0.69
brain	0.07	0.07	0.07	0.07	0.02	0.02	0.00	0.00	0.00	0.01	0.00	0.00	0.15	0.15
urine	25.90	15.88	4.21	1.73	4.72	3.62	0.73	1.16	0.19	0.19	1.09	1.28	15.75	5.41

Table S1. Ex vivo biodistribution data for all animal groups. Each group has two columns, showing %ID/g values for tissues, and their standard deviations (SD %ID/g). The last two columns refer to additional groups injected with [68Ga]KP46 (tracer level) and culled 2 h post injection, shown graphically in Fig. S8. SI = small intestine; LI = large intestine.

[^{68}Ga]KP46 biodistribution in *nu/nu* mice

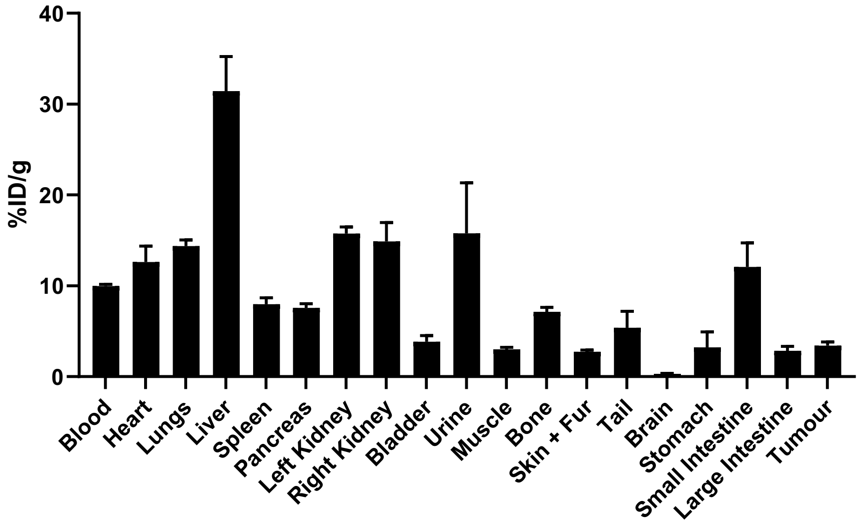

Figure S8. *Ex vivo* biodistribution of [^{68}Ga]KP46 (tracer level) in *nu/nu* mice bearing A375 xenografts at two hours post i.v. injection via tail vein. Minimal uptake was observed in tumours. Values are expressed as mean ± SD (n = 3). (See below for methods)

***Methodology for ex vivo* biodistribution 2 h after intravenous injection of [^{68}Ga]KP46**
Mice were intravenously injected with tracer level [^{68}Ga]KP46 and culled two hours post injection for organ harvesting and gamma counting. Consistent with the data shown in the main manuscript for 4 h post-injection, 31.4 ± 3.8%ID/g was seen in the liver two hours after the i.v. injection of [^{68}Ga]KP46. Activity was also observed in the kidneys (30%ID/g for both kidneys) and urine, indicating excretion through urinary system. Significant activity was also seen in the lungs, heart, and small intestine (14.3 ± 0.6, 12.6 ± 1.7 and 12 ± 2.6 %ID/g, respectively), with minimal activity accumulating in the tumour (3.4 ± 0.4%ID/g) (Figure S8).

^{69}Ga tissue analysis
To measure how much natural gallium was present in the tissue samples from mice in groups D and F, using ICP-MS, samples were left to decay at –20° C, then samples of liver, stomach, small intestine, large intestine, urine, blood, faeces, heart and tumour (after bulk tissues being sliced into small pieces) were transferred to 15 ml metal-free centrifuge tubes (Elkay Laboratories, 2086-500). Samples were digested by adding optima grade concentrated HNO_3 (67–68%, Fisher Scientific*)* (3–5 ml) and allowing to stand at room temperature for 24 hours. Samples were transferred to acid-cleaned 7–15 ml PTFE microwave digestor vessels and placed in a Milestone UltraWAVE microwave digestion system, heated to 220° C over the course of a 15-minute period and maintained at that temperature for a further 10 minutes to complete the digestion process. Sample aliquots of 0.5 ml were transferred to acid-cleaned trace metal grade HDPE centrifuge tubes (purchased from VWR) and diluted to 10 ml (20-fold) with purified water from an Elga PureLab purification system and then analysed by ICP-MS.

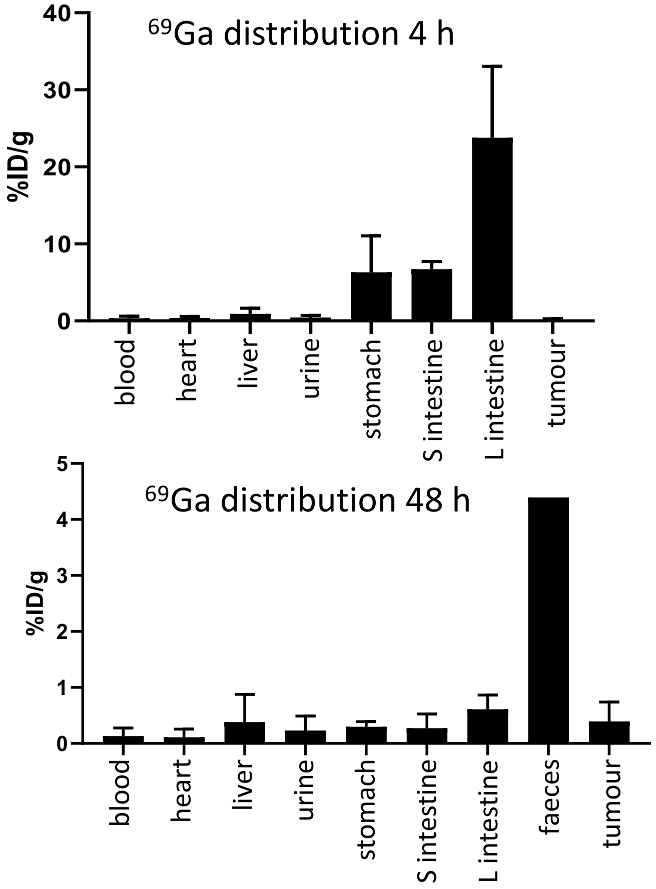

Figure S9. ICP-MS analysis of ^{69}Ga in tissue samples 4 h post oral administration of [^{68}Ga]KP46 (bulk, group D, top) and 48 h post oral administration of [^{67}Ga]KP46 (bulk, group F, bottom). All data are represented as mean ± SD (n = 4, except for faeces where n = 1). It was not possible to obtain analysable solutions from samples of bone by the method used. Biodistribution in analysable tissues was similar to the 4 h and 48 h distribution of ^{68}Ga and ^{67}Ga, respectively, with highest levels in faeces and GI tract (bone excluded).

[^{67}Ga]KP46 biodistribution

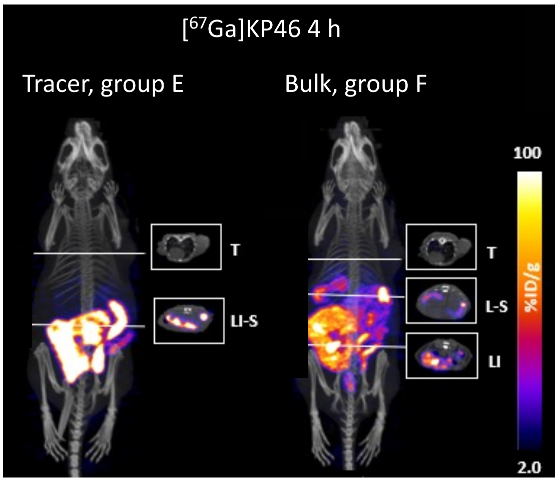

Figure S10. SPECT/CT images (maximum intensity projection, dorsal aspect) of nu/nu mice bearing A375 xenografts 4 hours post oral administration of [^{67}Ga]KP46 at tracer (left, group E) or bulk (right, group F) level. T = tumour, L = liver, S = stomach, LI = large intestine (exemplar from n = 4). At this time point the images show no significant difference between the bulk and tracer level distribution.

^{67}Ga citrate to ^{67}Ga chloride conversion method

^{67}Ga was provided as [^{67}Ga]Ga-citrate, typically 418-430 MBq in 5.5 ml at the time of receipt (76-78 MBq/ml). The ^{67}Ga-citrate solution was passed through a Sep-Pak plus light silica cartridge (part number WAT023537) at a flow rate of 1 ml/min to trap the ^{67}Ga on the silica cartridge. The eluate was passed through again to capture more (total 80%) of the available radioactivity on the cartridge. The cartridge was then washed twice with 5 ml of H_2O to remove citric acid, then eluted with 600 µl of 0.1 M HCl in 50 µl fractions. Fractions with the highest activity (fraction 4, 5, and 6) were used for experiments.

Tumour xenograft development

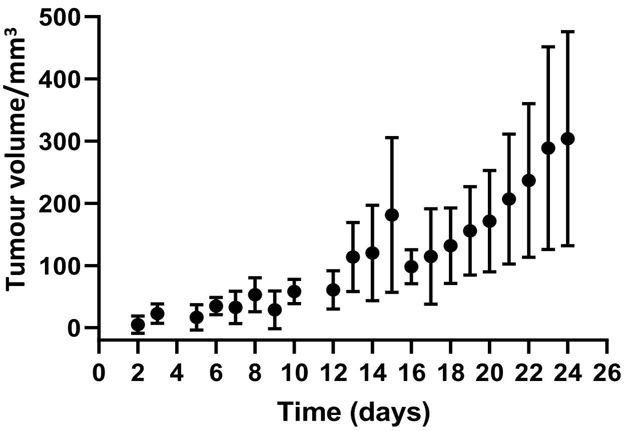

Figure S11. Tumour growth profile of A375 human melanoma cells in female athymic nude mice (2.5 x 10^6 cells). Data are reported as mean ± SD (n=24).

Developing a method for the oral administration of [^{68}Ga]KP46

In the first pilot study, mice bearing a tumour xenograft (A375) were anaesthetised, orally administered with [^{68}Ga]KP46 (10 MBq, 400 µl) and PET scanned (no CT scan was performed) for four hours (n = 1), or orally administered with [^{68}Ga]KP46 (1–2 MBq, 400 µl) and kept anaesthetised for four hours (n = 3), before being culled for organ harvesting and gamma counting. To enhance the gastrointestinal motility, in the second pilot study, healthy mice (n = 3) were anaesthetised, orally administered with [^{68}Ga]KP46 (6–8.5 MBq, 400 µl) and allowed to recover for three hours before being re-anaesthetised and PET-scanned for one hour. In both studies, mice were fasted for no more than 18 hours during the entire study.

PET imaging from the first pilot study group revealed that after oral administration, most of the radioactivity was in the stomach, with minimal uptake in the small intestine. The *ex vivo* biodistribution data confirmed the PET image results and showed 324.4 ± 75.6 %ID/g in the stomach (77.6 ± 31.7 %ID), with less activity seen in the small intestine (33.4 ± 8.3%ID/g, 30.07 ± 9.8 %ID). Tumours could not be localised in the PET images and from *ex vivo* biodistribution data, very low activity was observed in the tumour (0.6 ± 0.3%ID/g) (Figure S12). The low absorption and low translocation from stomach to small intestine of [^{68}Ga]KP46 indicates the negative effect of anaesthesia on gastrointestinal motility. However, following the addition of a three-hour recovery period prior to scanning, the PET images showed the most activity in the large intestine. *Ex vivo* data supported the PET scan results and showed less activity in the stomach (27.5 ± 43.2%ID/g, 5.07 ± 7.88 %ID) and more activity in the large intestine (62.3 ± 35.4%ID/g, 50.09 ± 19.6 %ID) compared to the first group. More activity was also noticed in the blood, liver and urine (Figure S12), indicating improved absorption. Due to the delayed gastrointestinal transit in the mice kept anaesthetised between administration and scanning at 4 h, the method involving keeping mice awake between administration and scanning was adopted for experiments reported in the main manuscript.

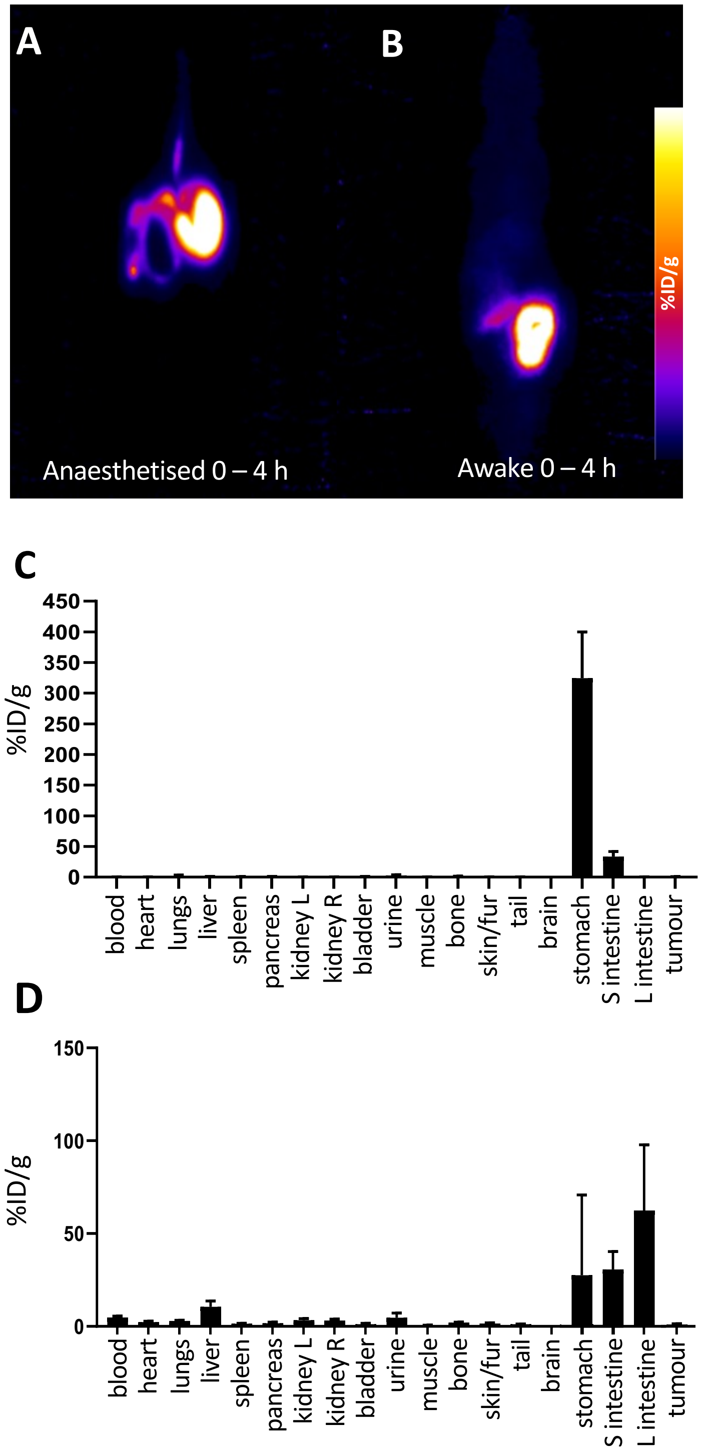

Figure S12. Development of oral administration scanning protocol. **A** and **B**: Exemplar PET image (maximum intensity projection, dorsal aspect) of mice 4 h after oral administration of [^{68}Ga]KP46 (tracer level), **A** with animals maintained under anaesthesia from administration to scanning, **B** with animals awake from administration to scanning. **C** and **D**: *ex vivo* biodistribution data for mice (n = 4) kept under anaesthesia and awake, respectively, between administration and scanning. Data shown are mean and standard deviation. Both scans and charts show that gastrointestinal transit is delayed by anaesthesia.
